# Monitoring the Electrochemical Failure of Indium Tin Oxide Electrodes via Operando Ellipsometry Complemented by Electron Microscopy and Spectroscopy

**DOI:** 10.1021/acsami.3c17923

**Published:** 2024-02-07

**Authors:** Alexey Minenkov, Sophia Hollweger, Jiri Duchoslav, Otgonbayar Erdene-Ochir, Matthias Weise, Elena Ermilova, Andreas Hertwig, Manuela Schiek

**Affiliations:** † Christian Doppler Laboratory for Nanoscale Phase Transformations, Center for Surface- and Nanoanalytics (ZONA), Johannes Kepler University, A-4040 Linz, Austria; ‡ Center for Surface- and Nanoanalytics (ZONA), Institute for Physical Chemistry (IPC) & Linz Institute for Organic Solar Cells (LIOS), Johannes Kepler University, A-4040 Linz, Austria; § FB 6.1 Oberflächenanalytik und Grenzflächenchemie, Bundesanstalt für Materialforschung und -prüfung (BAM), Unter den Eichen 44-46, D-12203 Berlin, Germany

**Keywords:** indium tin oxide, operando ellipsometry, cyclic voltammetry, electron microscopy and spectroscopy, solid–liquid interface

## Abstract

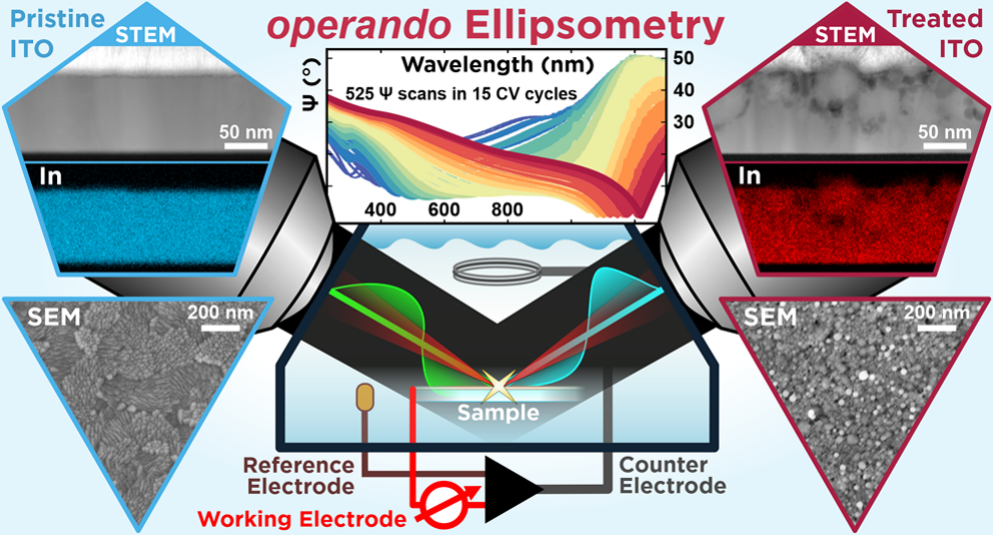

Transparent conductive oxides such as indium tin oxide (ITO) are standards for thin film electrodes, providing a synergy of high optical transparency and electrical conductivity. In an electrolytic environment, the determination of an inert electrochemical potential window is crucial to maintain a stable material performance during device operation. We introduce operando ellipsometry, combining cyclic voltammetry (CV) with spectroscopic ellipsometry, as a versatile tool to monitor the evolution of both complete optical (i.e., complex refractive index) and electrical properties under wet electrochemical operational conditions. In particular, we trace the degradation of ITO electrodes caused by electrochemical reduction in a pH-neutral, water-based electrolyte environment during electrochemical cycling. With the onset of hydrogen evolution at negative bias voltages, indium and tin are irreversibly reduced to the metallic state, causing an advancing darkening, i.e., a gradual loss of transparency, with every CV cycle, while the conductivity is mostly conserved over multiple CV cycles. Post-operando analysis reveals the reductive (loss of oxygen) formation of metallic nanodroplets on the surface. The reductive disruption of the ITO electrode happens at the solid–liquid interface and proceeds gradually from the surface to the bottom of the layer, which is evidenced by cross-sectional transmission electron microscopy imaging and complemented by energy-dispersive X-ray spectroscopy mapping. As long as a continuous part of the ITO layer remains at the bottom, the conductivity is largely retained, allowing repeated CV cycling. We consider operando ellipsometry a sensitive and nondestructive tool to monitor early stage material and property changes, either by tracing failure points, controlling intentional processes, or for sensing purposes, making it suitable for various research fields involving solid–liquid interfaces and electrochemical activity.

## 1 Introduction

Transparent electrodes are omnipresent in displays, optoelectronic, photovoltaic, and electrochromic devices, as well as in bioelectronic and photoelectrocatalytic applications. Among them, the transparent conductive oxide indium tin oxide (ITO) has been and still is the industrial and laboratory standard for more than half a century.^1−9^ Due to shrinking indium resources, there are multiple approaches to replace ITO;^10−12^ however, research on the optimization of ITO electrode layers is still ongoing.^13−19^ Despite the multifarious presence of ITO in research and in the literature, studies on degradation are rather scarce. Especially for the utilization in bioelectronics and photoelectrocatalysis purposes, contact with an electrolytic environment demands the characterization of an inert electrochemical potential window.^20^ Since ITO is already in an oxidized form, the positive potential window is large.^21^ However, multiple cathodic reduction pathways at negative potential are possible depending on the electrolyte specifications, where reductive degradation is promoted under acidic conditions and rather inhibited in a basic environment.^22−27^ Acidic chemical degradation has been reported even at solid interfaces.^28,29^ Typically, this leads to a visible darkening of the ITO layer caused by the reductive formation of metallic indium and tin nanoparticles and, finally, a loss of electrical conductivity. Darkening due to such reductive loss of oxygen is also known for other functional oxidic materials, for instance during field-assisted sintering^30−32^ or calcination/annealing,^33,34^ and it may even be beneficial because it widens the spectral response of photocatalytic electrodes^35,36^ or oxygen vacancies enhance the ionic conductance of ceramics.^30^

Monitoring of electrochemical processes can be obtained by so-called operando approaches. Such methods are established in electrocatalysis, battery, and bioelectrochemistry research, and they describe the spectroscopic or microscopic monitoring of electrochemical processes under operational conditions.^37−46^ Among them, spectroelectrochemistry relies on the optical transparency of the working electrode,^47^ and with the implementation of ITO electrodes, this method was commenced to study redox-active proteins about 60 years ago.^48^ Up to now, the operando combination of electrochemistry and spectroscopic ellipsometry is rather rarely applied, possibly due to the challenges of optical modeling and the limitations of continuous and reasonably smooth layers.^49−56^ Nonetheless, ellipsometry is a powerful tool to obtain both the dielectric optical response function (i.e., complex refractive index) of thin films as well as the electric properties for Drude-like behavior, which apply well to ITO thin films.^57−59^ Furthermore, ellipsometry is a sensitive probe to monitor the adsorption of ultrathin layers at solid–liquid interfaces such as self-assembled monolayers and biomolecules.^60−64^ Even specific immobilization of biomolecules and cells at functionalized hybrid interfaces for biosensing purposes can be traced.^65−67^ More specifically, operando ellipsometry allows to study reversible ion intercalation processes upon electrochemical cycling in battery-related research.^52−54^ For spatially resolved investigation, operando imaging ellipsometry is an emerging probe for battery electrodes, and it provides insights by monitoring the local change of ellipsometric parameters at selected wavelengths.^68,69^

**Figure 1:**
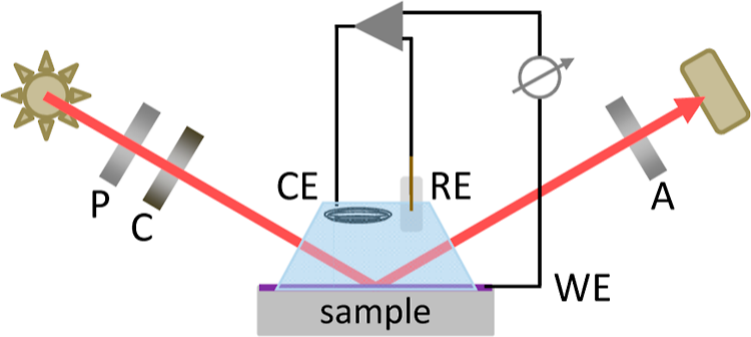
Operando approach combines spectroscopic ellipsometry in reflection with electrochemistry. The ellipsometric probe beam enters and exits the electrochemical cell filled with a water-based electrolyte (here, physiological HEPES-buffered Krebs–Ringer solution) under a fixed angle of incidence (AOI = 70°), hitting the cell windows under normal incidence. The cell is mounted on top of the flat-lying sample, and a rubber seal defines an active area of (0.85 ± 0.05) cm^2^ on the sample. A three-electrode electrochemical setup (IVIUM CompactStat potentiostat) is implemented for cyclic voltammetry (CV) cycling with a coiled platinum wire as a counter electrode, a chlorinated silver thread as a pseudo-reference electrode, and the sample (here, ITO) as a working electrode. Electrochemical cycling is synchronized with the single rotating compensator ellipsometric measurements (J.A. Woollam M-2000 DI; P = polarizer, C = compensator, and A = analyzer) probing the parameters Ψ and Δ suitable for isotropic and uniaxial samples within the spectroscopic range from 225 to 1300 nm according to the transparency window of the electrolyte. The CCD-based detection provides with 2.63 s fast enough acquisition of Ψ and Δ spectra to measure full spectra at least every 0.15 V step for a typical CV scan rate of 50 mV/s.

In this work, we present operando spectroscopic monitoring of the optical and electrical properties of sputter-coated ITO layers by ellipsometry during electrochemical cycling in a physiological electrolyte. In Figure 1, our operando setup is sketched. We have chosen an aqueous physiological electrolyte having a neutral pH to relate to potential bioelectronic applications, such as electrochemical biosensors, photobioelectrodes to study natural and artificial photosystems or for biophotovoltaic or bioelectrocatalytic purposes, and ultimately bioelectronic medical interfaces, more specifically photocapacitors for stimulation of neuronal cells.^4,6,7,70−79^ For the latter, ITO is rather used as an additional coating layer for ultrathin gold electrodes to prevent anodic chloride-mediated corrosion and cathodic electrolytic activity even in physiological electrolytes, as well as to serve as an adhesion layer for biopolymers and cells without sacrificing transparency.^79^ All applications rely on both the conductivity and the full spectral transparency of ITO electrodes to allow for steady transmission of probing or stimulating light beams. To avoid that, for instance, incipient darkening of the ITO electrode is mixed up with the signal from an analyte of a biosensor, precise awareness of the ITO’s passive operational electrochemical potential window for a specific electrolyte environment is crucial. While the reductive degradation mechanism is basically the same in different aqueous electrolytes, the onset potential depends on pH, shifting to more positive potentials for decreasing pH, as well as on dissolved redox-active electrolyte components, which can also have an impact on the reduction onset potential.^20,25^

In this exemplary study, we observe that the optical darkening of the ITO layer progressively sets in before the conductivity deteriorates. The change in optical appearance happens stepwise and irreversibly during chemical reduction and the loss of oxygen within a negative bias interval. Apart from commercial ITO samples on glass substrates, we have also prepared ITO films on silicon wafers with thick insulating silicon dioxide layers to perform reliable post-operando cross-sectional transmission electron microscopy (TEM) complemented with energy dispersive X-ray spectroscopy (EDXS) for deeper morphological and chemical insights. By this, we reveal that the initially polycrystalline but smooth and homogeneous ITO layer is disrupted by the reductive formation of metallic nanodroplets. Due to this excessive surface roughness formation, the modeling of ellipsometric data is stretched to its limits. However, monitoring the spectroscopic ellipsometric parameters is still valuable to trace the changes in optical and electrical sample parameters during multiple electrochemical CV cycles. We employ the ITO-physiological electrolyte solid–liquid interface as an intuitive model sample to demonstrate the operando ellipsometry method.

## 2 Results and Discussion

**Figure 2:**
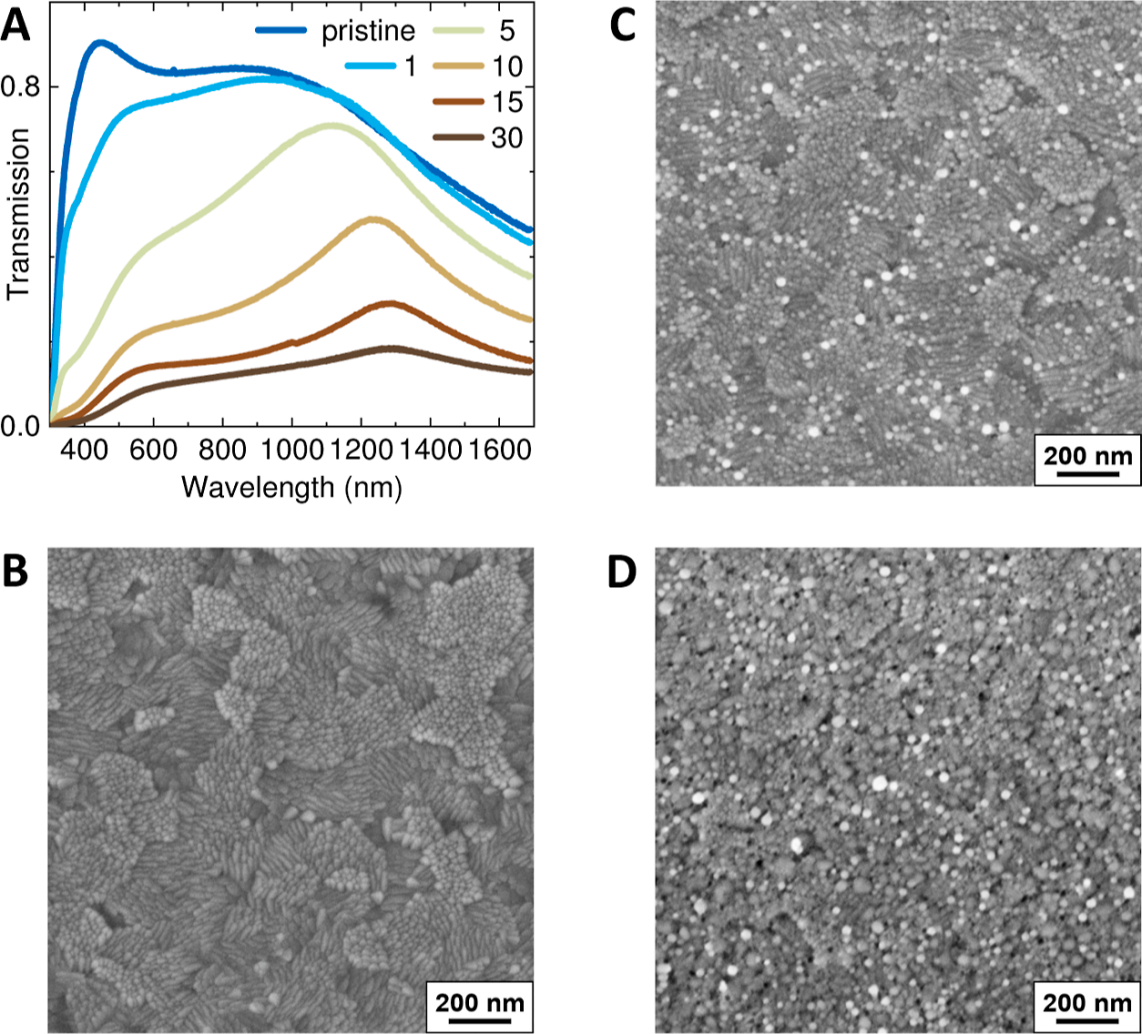
(a) Transmission spectra of a commercial ITO-glass sample before and after electrochemical cycling in HEPES-buffered Krebs–Ringer electrolyte, potential range starting from 0 V down to −1.5 V and up to +0.8 V measured against a Ag/AgCl pseudo-reference electrode with a scan rate of 50 mV/s. Number of cycles is indicated in the legend. Treated samples were rinsed with deionized water and blow-dried with nitrogen steam. Plan-view SEM images of ITO-glass samples: (b) pristine material, (c) after 1 cycle comprising round-shaped nanoparticles with a median size of approximately 25 nm, and (d) after 15 cycles with nanoparticles of approximately 30 nm median size.

To introduce the problem of ITO degradation due to electrochemical cathodic reduction, representative transmission spectra and scanning electron microscopy (SEM) images of commercial ITO layers on glass substrates (ITO-glass) are shown in Figure 2 before and after electrochemical cycling in the HEPES-buffered Krebs–Ringer electrolyte. A pristine ITO-glass sample has high transparency exceeding 80% transmission within the visible spectral range, while within the near-infrared range, a Drude-like free carrier absorption decreases the transparency;^57^ see Figure 2a dark blue line. The SEM image in Figure 2b shows the typical morphology of an untreated polycrystalline ITO layer consisting of elongated grains organized in randomly oriented domains with a few to several tens of nanometer lateral dimensions. The random, polycrystalline nature is also confirmed by X-ray diffraction, as shown in the Supporting Information in Figure S1. Electrochemical cycling is conducted in a potential bias range starting from 0 V down to −1.5 V and up to +0.8 V measured against a Ag/AgCl pseudo-reference electrode with a scan rate of 50 mV/s. The transmission spectra in Figure 2a of a pristine ITO-glass sample and after 1, 5, 10, 15, and 30 cycles show that the transmission gradually decreases, starting already after a single electrochemical cycle. Thus, this potential window offers a suitable dynamic range to study the gradual irreversible electrochemical degradation by operando ellipsometry. The ITO layer remains intact at negative potentials down to −1.0 V against Ag/AgCl in the neutral pH physiological electrolyte (data not shown). The onset of irreversible degradation is between −1.2 and −1.3 V against Ag/AgCl (data not shown), which is in agreement with other studies using mildly acidic electrolytes.^25^ A harsh biasing down to −2.0 V rapidly leads to the complete destruction of the ITO layer, after only 4 CV cycles, the sample has turned nonconductive. CV and operando ellipsometry measurements are shown in the Supporting Information in Figures S2 and S3 for an ITO-glass and an ITO-wafer sample, respectively. The drastic cross-sectional morphology evolution of this specimen is documented in Figure S4.

Plan-view SEM was performed for treated ITO-glass after 1 and 15 CV cycles within the chosen dynamic range. In Figure 2c, round-shaped nanoparticles with a median size of approximately 20 nm can be seen, which were formed due to the reduction of ITO during 1 cycle already. Complementary X-ray photoelectron spectroscopy (XPS) survey spectra of the pristine and treated specimens can be found in the Supporting Information in Figure S5. The formation of indium droplets on the surface is in agreement with other studies on the electrochemical stability of ITO.^24−26^ Interestingly, underneath nanoparticles, the pristine polycrystalline ITO structure looks preserved, which ensures retained film conductivity. The change influences only a relatively thin surface layer, which instantly reduces the transparency. The SEM image of the specimen after 15 cycles shown in Figure 2d reveals a noticeable morphology alteration. Although the size of the observed nanoparticles remains virtually unchanged (30 nm on average) after longer electrochemical treatments, their number increases significantly. The characteristic polycrystalline structure also appears to have been destroyed. The initially continuous layer now contains many pores, which finally lead to an irreversible deterioration in material conductivity.

### 2.1 Ambient Ellipsometry

**Figure 3:**
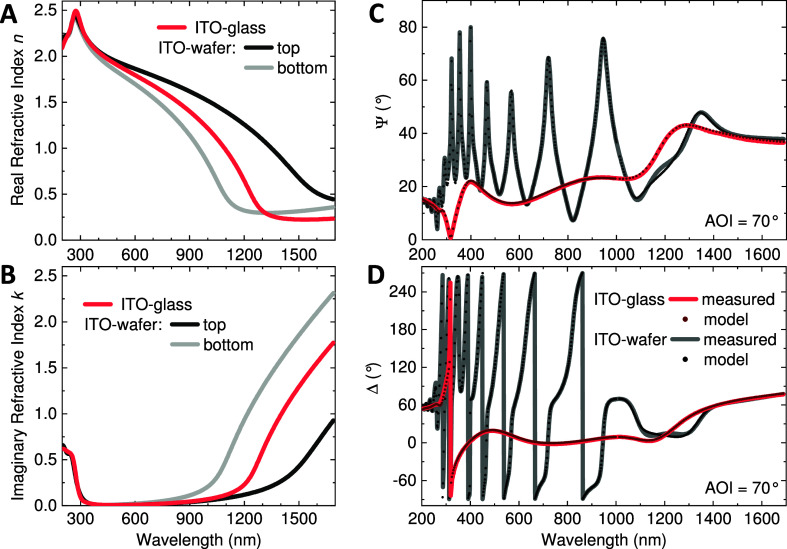
Real and imaginary part, *n* and *k*, of the complex refractive indices for pristine ITO-glass (red lines) and ITO-wafer (black and gray lines) samples as a result of a MSA are shown in (a,b), respectively. Note that the ITO-wafer samples comprise a graded ITO layer, i.e., a different complex refractive index at the top (black line) and the bottom (gray line) of the layer with a linear, gradual vertical change. In (c) the Ψ and in (d) the Δ spectra for AOI = 70° in ambient conditions are plotted for an ITO-glass (red line) and an ITO-wafer (gray line) sample. Solid lines are measured data and dotted lines represent the modeling result. Note the interference pattern for the ITO-wafer sample, which is due to coherent reflections from the thick SiO_2_ layer.

The complex refractive index of both pristine ITO-glass and ITO-wafer samples was determined by ellipsometry under ambient conditions. The data shown in Figure 3a,b are results from a multisample analysis (MSA) performed with CompeteEASE version 6 to obtain representative and reliable values for the refractive index. For ITO-glass transmission intensity spectra are included for parameter decorrelation.^80,81^ Please read details about the fitting procedure in Subsection 4.2. Briefly, an isotropic oscillator model composed of a Tauc–Lorentz and a Gaussian oscillator plus a Drude term is used to describe interband transitions within the UV spectral range and to account for the free carrier infrared absorption, respectively, plus an effective medium surface roughness layer. For the ITO-wafer samples, a linear parametric grading of about 50% of the Drude term accounts for the changing resistivity of the layer from bottom to top, which entails a varying complex refractive index through the layer depth.^81,82^ The real part *n* of the refractive index is higher (lower) at the top (bottom) of the layer, Figure 3a black = top and gray = bottom line, respectively, and the imaginary part *k* behaves vice versa, Figure 3b. This resistivity grading can be reasoned with a material gradient, which is a varying In/Sn ratio at the top and bottom of the pristine film. This is clearly identified by EDXS of a TEM lamella, as discussed in Subsection 2.3. In general, the resulting resistivity of ITO layers is a complex interplay of processing parameters and chemical element ratios (especially oxygen vacancies),^83−85^ and its reasoning is beyond the scope of the present manuscript. The layer thickness *d* and resistivity ρ_(*E*)_ derived from ellipsometry, together with the resistance measured by 4-wire sensing, are summarized in Table 1. The commercial ITO-glass samples are slightly more conductive than the self-produced ITO-wafer samples, but for both, the resistivity is typical on the order of 10^–4^ Ω cm. Ellipsometric modeling and 4-wire sensing agree well; however, ellipsometry finds systematically slightly higher resistivities. The stated resistivity from ellipsometry is an effective value, and the given standard deviation is a result of averaging over effective values from different samples or measurements. In the case of the ITO-wafer samples, the resistivity is subjected to a parameter grade linearly increasing by roughly 50% from the bottom to the top of the layer. The effective resistivity is calculated using a parallel resistor model for the (here, 11) sublayers of each measurement; see eq 1 in Subsection 4.2.

**Table 1:** Mean Thickness and Resistivity from Ellipsometric Fitting ρ_(*E*)_ According to Equation 1 and Resistivity ρ_(4W)_ According to Equation 2 (See Subsection 4.2) of the ITO Layer for All Samples^a^

	ITO-glass^I^	ITO-wafer^II^
thickness *d* (nm)	110 ± 1	88 ± 1
4-wire sensing		
measured resistance *R*_S_ (Ω)	2.9 ± 0.1	5.6 ± 0.3
4-wire sensing		
resistivity ρ_(4W)_ (10^–4^ Ω cm)	1.4 ± 0.2	2.3 ± 0.4
ellipsometry		
resistivity ρ_(*E*)_ (10^–4^ Ω cm)	1.7 ± 0.2	2.9 ± 0.4

a The resistivity parameters from 4-wire sensing and ellipsometry are correlated because the thickness determined from ellipsometry is used for calculating both. The standard deviation results from averaging over multiple samples and multiple measurements (*N* = 6 for 4-wire sensing). The ρ_(*E*)_ for the ITO-wafer sample is an effective value subjected to linear parametric grading (11 sublayers) with roughly 50% higher resistivity at the top compared to the bottom of the layer (*N* = 12). ^I^ITO on glass sample plates type XY15S were bought from Xinyan Technology LTD. ^II^Sputter-coated ITO on (111) Si-wafer thermal oxide (1009 ± 1) nm.

The measured (lines) and modeled (dots) Ψ and Δ curves to obtain the complex refractive index of the ITO layers under ambient conditions are shown in Figure 3c,d for a selected angle of incidence (AOI = 70°). For the ITO-glass sample, the dark red model dots fully match the measured red lines, indicating a good fit to the data. This is in agreement with the low overall MSE of about 11 for the MSA, including transmission intensity spectra. The MSE is about 35 for the ITO-wafer samples, which is still reasonable but certainly higher. The black model dots do not fully match the measured gray lines within the spectral range from around 1050 to 1300 nm, indicating an inferior fit at the onset of the free carrier’s Drude tail. This is likely correlated with the resistivity grading of the self-produced ITO-wafer samples. However, a characteristic feature of the ITO-wafer’s Ψ and Δ spectra is the pronounced interference pattern caused by multiple coherent reflections within the transparent SiO_2_ interlayer present for insulating purposes. These overlaid features make the change in Ψ and Δ caused by ITO degradation during operando measurements less intuitive to capture by eye. Therefore, we present a detailed representative operando analysis for a single electrochemical cycle of an ITO-glass sample, as shown in Subsection 2.2.

### 2.2 Operando Ellipsometry

**Figure 4:**
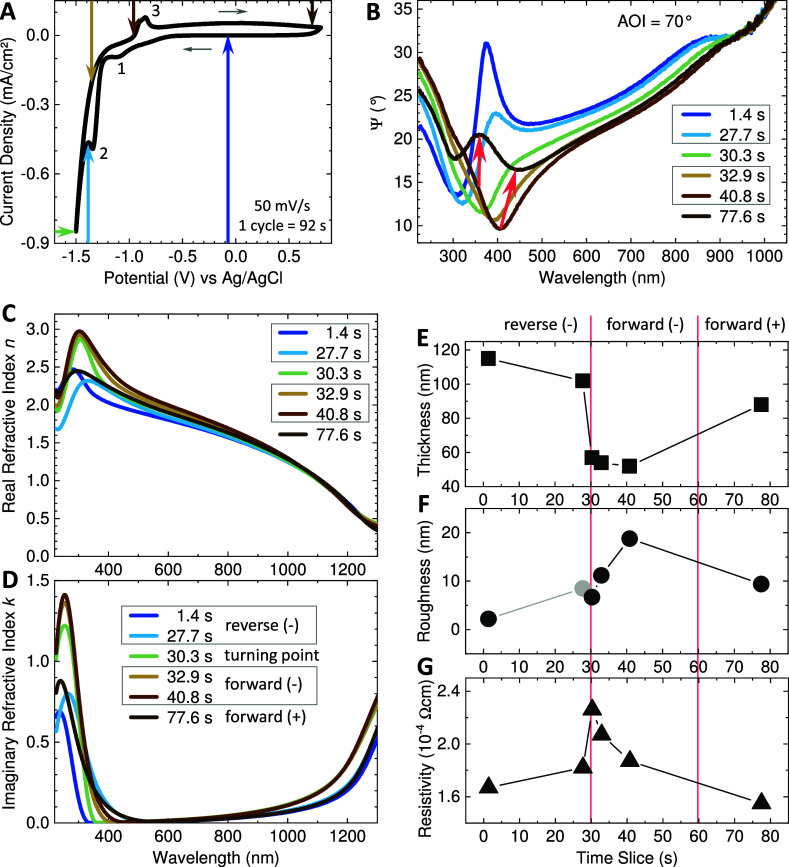
(a) Single-cycle CV of an ITO-glass sample (*N* = 1) in Krebs–Ringer electrolyte starting at 0 V down to −1.5 V and up to 0.8 V measured against a Ag/AgCl pseudo-reference electrode with a 50 mV/s scan rate. CV scan is completed in 92 s. Thin gray arrows indicate scan direction, and the numbers 1, 2, and 3 mark characteristic electrochemical reaction points. Sample area exposed to electrolyte was (0.85 ± 0.05) cm^2^. For the positions marked with colored arrows, ellipsometric Ψ curves at AOI = 70° in the electrolyte are plotted in (b). Selected time slice for each Ψ scan is given in the legend. Ψ scans were recorded every 2.63 s, giving 35 spectra per CV cycle. The red arrows in (b) illustrate the gradual change in Ψ value from time slice 40.8 to 77.6 s, scans in between are omitted for readability. A video clip showing synchronized, animated CV cycle and Ψ scans is included in the Supporting Information as Movie S1. The real and imaginary refractive indices *n* and *k* obtained by ellipsometric modeling are shown for the selected time slices in (c,d), respectively. Sample parameters for a time slice are extracted from the ellipsometric model to illustrate trends: (e) ITO layer thickness, (f) roughness, and (g) resistivity. Connecting lines are sketched to guide the eye. Light gray circle in (f) indicates that the absolute value of roughness is given. Red vertical lines divide the time scale for correlation with the CV bias regimes reverse negative, forward negative, and forward positive.

In Figure 4a, a single CV cycle of an ITO-glass sample in the physiological electrolyte is shown in (a), and for the six positions indicated with arrows at different potentials, the corresponding ellipsometric Ψ scans in the electrolyte are plotted in (b). Here, the point in time of recording, the time slice, labels the Ψ spectrum. With a scan rate of 50 mV/s, the CV cycle starting from 0 V down to −1.5 V and up to +0.8 V and back to 0 V takes 92 s to be completed. Ψ scans are recorded every 2.63 s giving 35 spectra for this single CV cycle. The AOI = 70° is fixed as given by the electrochemical cell. For a better representation of the simultaneous voltage sweeping and ellipsometric scanning, a video recording of animated plots can be found in the Supporting Information as Movie S1.

Three characteristic electrochemical reactions are indicated by numbers in Figure 4a. The first shallow dip (1) in reverse bias at around −1.1 V indicates the (full or partial) reduction of indium and tin oxide, which is at least partially reversible. The double peak (3) in forward bias around −0.9 V marks the corresponding oxidation of (metallic or lower-valent) indium and tin species.^20^ The dip (2) appears only for electrochemical cycling in ambient atmospheric conditions, i.e., in the presence of dissolved oxygen in the electrolyte. It indicates oxygen to peroxide reduction within the electrolyte.^86^ The absence of this reaction point is validated by a CV scan under nitrogen atmosphere conditions, as shown in the Supporting Information in Figure S7. Note that the absence of oxygen does not prevent the degradation of ITO since it follows a reductive mechanism. Irreversible reduction to metallic In and Sn occurs during the regime of hydrogen evolution marked by the sharp increase of current flow for negative potentials exceeding −1.3 V.

The Ψ scan recorded at a time slice of 1.4 s just after starting the CV cycle (dark blue arrow in Figure 4a) is plotted in dark blue in Figure 4b. It looks different from the Ψ spectrum of ITO-glass recorded in ambient conditions, as shown in Figure 3c, due to the water-based environment above the ITO layer and below the glass substrate. This water-based environment is included in the model, which otherwise is basically the same isotropic, nongraded model with a surface roughness layer as used for the ITO-glass in ambient environment, to obtain the complex refractive index. Screenshots of the CompleteEASE modeling showing all fit parameters as well as measured and fitted Ψ and Δ curves for the selected time slices are collected in Figure S8 in the Supporting Information. The real and imaginary parts of the complex refractive index for all selected time slices are shown in Figure 4c,d, respectively. The refractive index for the first time slice in the electrolyte (dark blue lines) is the same as the refractive index in ambient conditions just as expected; see red lines in Figure 3a,b, for comparison. Interestingly, the reversible reduction reaction (shallow dip (1) in the CV curve) does not impact the optical properties of the ITO layer. The Ψ spectrum and the refractive index corresponding to (1) remain unchanged and, therefore, are omitted in the graphs. The first change of Ψ and refractive index occurs at time slice 27.7 s in reverse bias at −1.38 V just after the reduction of dissolved oxygen within the electrolyte (dip (2) in CV scan); see light blue arrow and lines in Figure 4a–d. The absorption edge is slightly red-shifted more into the visible range and marginally increased, which indicates a visible coloring. The most pronounced change happens at time slice 30.3 s, which is at the potential turning point at the most negative bias of −1.5 V in the CV scan; see green arrow and lines in Figure 4a–d. The initial peak in Ψ at around 370 nm exceeding 30° turns into a dip going down to almost 11°. Both *n* and *k* clearly increase, basically maintaining the red-shifted absorption edge. The change in Ψ and refractive index progresses stepwise with the same trend also after turning into forward bias for the time slices 32.9 s (−1.35 V in CV scan) and 40.8 s (−0.95 V in CV scan), ocher arrow and lines, and light brown arrow and lines in Figure 4a–d. The Ψ value further decreases to less than 10° and red-shifts to 400 nm. This is expressed in the refractive index as a further increase in the extinction coefficient *k* and as a broadening of the peak within the real refractive index *n*. This would be noticeable by eye as a progressive darkening of the ITO layer. For continued forward bias sweeping into the positive potential range up to 0.8 V, a gradual, partial back-development in Ψ happens, which is indicated by the red arrows in Figure 4b, connecting the Ψ spectra for time slice 40.8 s (light brown line) and time slice77.6 s (dark brown line). In the refractive index, this is expressed in a drop in both *n* and *k* while maintaining the red-shifted absorption edge. This means that the ITO layer retains its coloring. With this, the ITO advantage of having a high optical transparency is no longer valid to its full extent after only a single CV cycle.

To allow further interpretation of the ellipsometry data, selected parameters are extracted from the modeling, which are ITO layer thickness, ITO surface roughness, and ITO resistivity; see Figure 4e–g, respectively. Note that these parameters serve to indicate trends only and should not be overinterpreted. Especially the surface roughness is a rather abstract parameter, being an effective medium approximation that statistically mixes the ITO bulk refractive index with 50% of voids (air refractive index), thereby creating a surface layer with a lower refractive index. However, the thickness of the surface roughness layer can turn negative, indicating an increased refractive index of the surface layer. Thickness is the only fit parameter, and it takes half of its thickness from the bulk ITO layer below. From the parameters given in Figure 4e–f, the following can be estimated: during reverse bias, the ITO layer thickness decreases (black squares in (e)), while the resistivity increases (black triangles in (g)). The surface roughness turning negative (gray circle in (f)) hints at an initial densification of the surface layer sweeping from zero bias down to −1.38 V (time slice 27.7 s). Reaching the potential turning point of −1.5 V at time slice 30.3 s, the layer thickness suddenly drops by about half its thickness (black square in (e)), accompanied by a clear rise of resistivity (black triangle in (g)) and an increased, positive surface roughness (black circle in (f)). This can be interpreted as a loss of material: molecular oxygen is released into the electrolyte upon the reductive formation of metallic In and Sn. Indium and tin can, to some extent, also be released in ionic, only partially reduced form into the electrolyte. Metallic nanodroplets are forming on the surface, giving it a certain surface roughness. During forward sweep but yet within the negative bias range (time slices 32.9 and 40.8 s), the loss of material, including the possible formation of pores near the surface and the formation of metallic droplets carries on. This can be deduced from the further slight decrease in layer thickness (black squares between the red vertical lines in (e)) and the clear increase in surface roughness (black circles in (f) between the vertical red lines). The resistivity slightly lowers again (black circles in (f) between the vertical red lines), which could point to a contribution of the surface metallic droplets to the overall conductivity. During the forward sweep from time slice 40.8 s up to time slice 77.6 s (+0.8 V), the layer thickness increases again to about two-thirds of the original value (black square (e)), the surface roughness lowers to about half of its maximum value (black circle (f)), and the resistivity decreases to an even slightly smaller value than the starting resistivity (black triangle (g)). This is indicative of replating indium and tin from solution or partial reoxidation of metallic In and Sn. An increased share of metallic or lower-valent In and Sn at the surface might be beneficial for the electrical performance at this stage. Furthermore, the fact that modeling of the treated ITO layer with a stable bulk ITO layer plus a surface layer reveals that the electrochemical degradation initially happens at the surface of the material, although progressing down to the bottom over time, while the bottom of the layer retains its properties for a while. This is also corroborated by a cross-sectional TEM investigation, which has been conducted on an ITO-wafer sample after 15 CV cycles and is discussed in Subsection 2.3.

**Figure 5:**
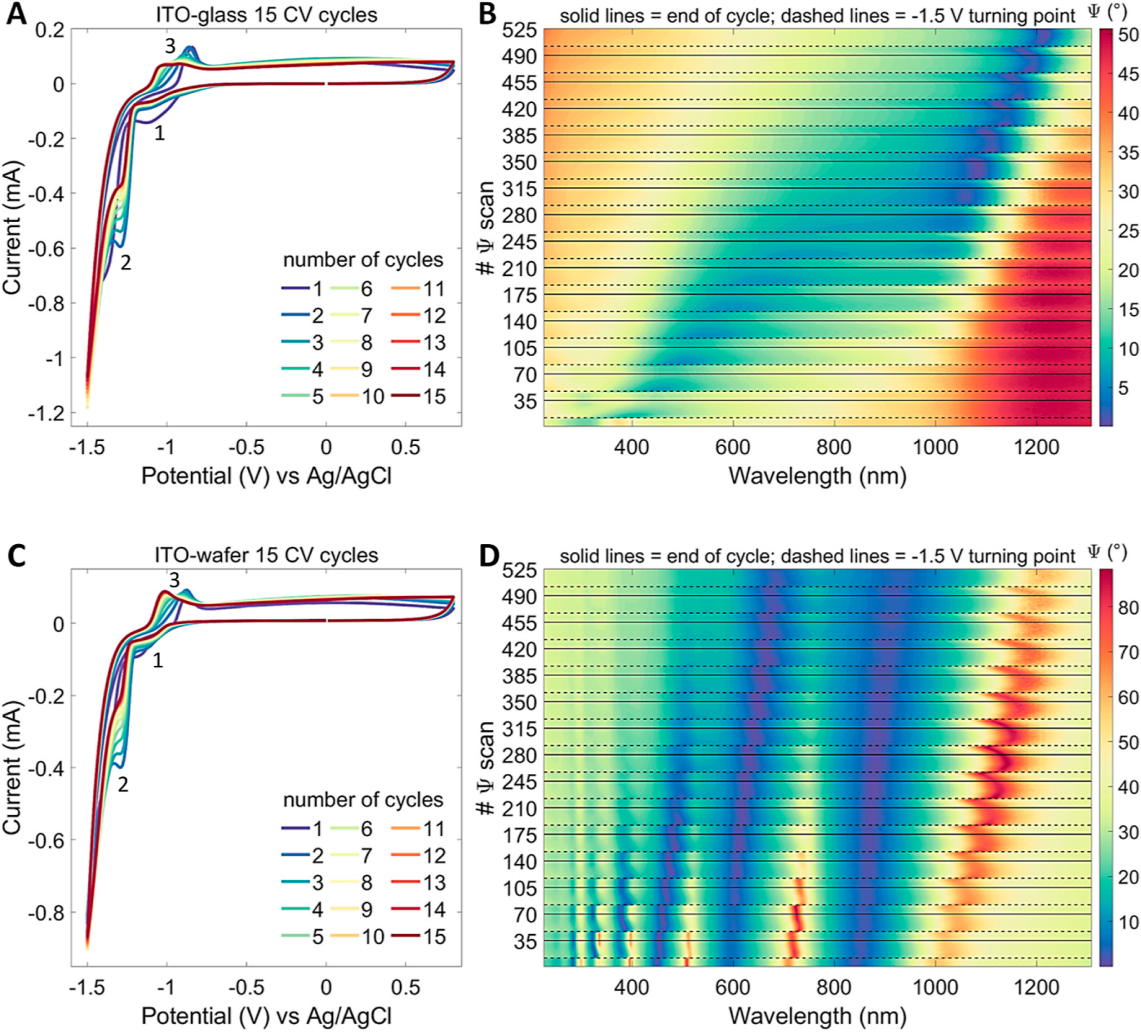
Fifteen CV cycles in pH-neutral Krebs–Ringer electrolyte starting from 0 V down to −1.5 V and up to +0.8 V and back to 0 V measured against a Ag/AgCl pseudo-reference electrode with a 50 mV/s scan rate, giving 92 s per cycle: ITO-glass sample (*N* = 1) (a,b) and ITO-wafer sample (*N* = 1) (c,d). CV scans are plotted in (a,c), while (b,d) show 525 ellipsometric Ψ spectra (35 per CV cycle) recorded every 2.63 s in the electrolyte at AOI = 70° as two-dimensional contour plots. Dashed lines indicate the potential turning point from reverse to forward bias at −1.5 V, and the solid lines mark the end of a CV cycle at 0 V. Sample area exposed to electrolyte was (0.85 ± 0.05) cm^2^. Video clips showing synchronized animated CV and Ψ scans gathered for the ITO-glass and ITO-wafer samples are included in the Supporting Information as Movies S2 and S3, respectively.

Electrochemical operando cycling with 15 CV cycles in the potential window from −1.5 to +0.8 V is shown in Figure 5 for an ITO-glass sample in the upper row and for an ITO-wafer sample in the lower row. The CV scans are shown in (a) and (c) and the corresponding 525 Ψ spectra are plotted in a two-dimensional representation in (b) and (d). Here, the solid lines indicate the end of a CV cycle, and the dashed lines mark the turning point from reverse to forward bias at −1.5 V of the potential sweep. The Ψ spectra of the ITO-wafer sample in (d) exhibit more features because the optical response from the ITO-layer is convoluted with interference patterns caused by multiple coherent reflections within the transparent SiO_2_ interlayer, which is required for electrical insulation from the Si substrate. For a better representation of the simultaneous voltage sweeping and ellipsometric scanning, animated CV and Ψ plots can be found in the Supporting Information as Movies S2 and S3 for the ITO-glass and ITO-wafer samples, respectively. We refrain from modeling ellipsometry spectra beyond a single CV cycle. The disruption of the surface by the progressive formation of metallic nanodroplets, as evidenced by electron microscopy severely impairs the interpretation of simple Ψ and Δ spectra. Random scattering causes depolarization of the reflected beam, which stretches ellipsometry as a polarization-sensitive method to its limits.^87^ Nonetheless, there are still lessons to learn from the operando ellipsometric parameter monitoring.^68,69^ For both ITO-glass and ITO-wafer samples, a “jump” in the Ψ spectra is obvious at every potential turning point; see dashed lines in Figure 5b,d. This is followed by a gradual, but never full restoration of Ψ value until the end of a voltage cycle returning to 0 V, see solid lines in (b) and (d). The “recovery” even proceeds to some extent until the next potential turning point. With this, the electrochemical degradation of ITO is progressively leaping forward with every CV cycle within the negative bias range. The degradation affects basically the optical properties, i.e., the transparency of the ITO layer is gradually lost. See also the transmission spectra recorded post-operando in dry conditions plotted in Figure 2a directly showing the progressive loss of transparency.

On the contrary, the purely electrical properties of ITO remain stable over 15 CV cycles, which is reflected by the repeatable CV scans, as given in Figure 5a,c. The same holds true for even 30 CV cycles (data not shown). The area exposed to the electrolyte is the same for both samples, defined by the electrochemical cell to be (0.85 ± 0.05) cm^2^. This means that the ITO-glass sample shown in (a) carries a marginally larger amount of overall current. Both samples exhibit the same three characteristic reaction points indicated by consecutive numbers 1, 2, and 3 in the sweeping direction next to the CV curves. The shallow dip (1) in reverse bias around −1.1 V indicates the (full or partial) reduction of indium and tin oxide, which is at least to some extent reversible. The double peak (3) in forward bias marks the corresponding oxidation of (metallic or lower-valent) indium and tin species.^20^ This double peak evolves with repeated electrochemical cycling: initially, the maximum is at around −0.9 V, and with an increasing number of CV cycles, the shoulder at around −1.0 V grows at the expense of the initial peak. The growth of the shoulder peak is more pronounced for the ITO-wafer sample. The dip (2) indicates the oxygen to peroxide reduction of dissolved molecular oxygen within the electrolyte,^86^ see also Figure S7 in the Supporting Information. It becomes less pronounced with repeated CV cycling. The sharp increase in current flowing at more negative potentials is due to the hydrogen evolution reaction, which causes the irreversible reduction to metallic In and Sn, leading to the permanent darkening of the ITO layer.^22,27^

### 2.3 Post-Operando Microscopy and Spectroscopy

**Figure 6:**
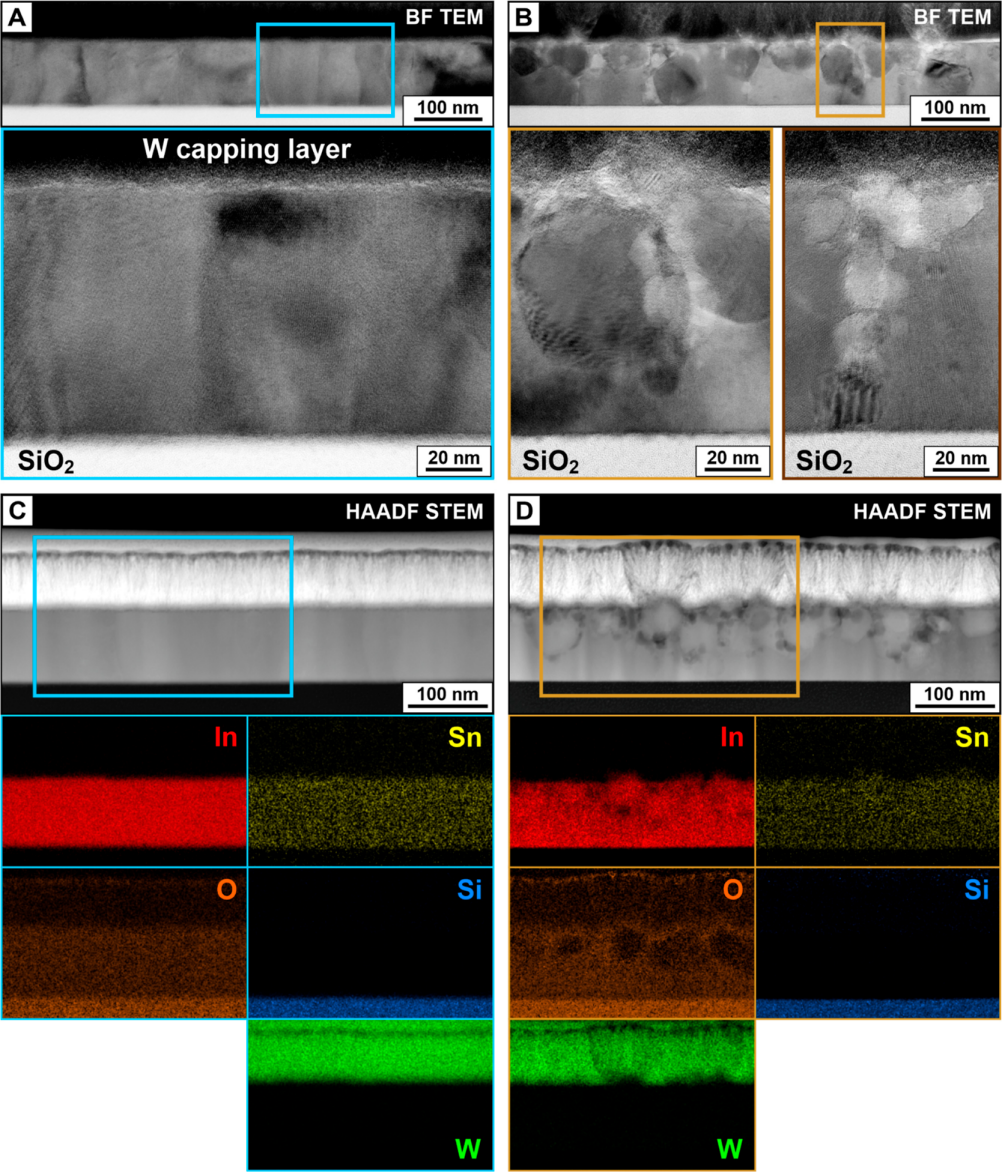
TEM investigation summary of an ITO-wafer sample. (a) Bright-field (BF) TEM image of the pristine ITO layer with a high-resolution snapshot of the region highlighted with a blue frame showing a columnar polycrystalline structure. (b) BF TEM image of the treated specimen (15 CV cycles) reveals the formation of distinct nanoparticles predominantly in the top part of the layer. High-resolution images (brown frames) represent the appearance of voids around particles. HAADF STEM imaging and corresponding EDXS elemental maps of pristine (c) and treated (d) ITO. W maps show a capping layer deposited to protect the surface during FIB-assisted lamella preparation.

To evaluate the morphological and chemical evolution of the ITO layer caused by electrochemical treatment, we take advantage of cross-sectional TEM characterization, including EDXS mapping. From this, we obtain the specimen’s cross-sectional elemental profile with spatial resolution on a nanometer scale. Note that conventional ITO-glass samples are not suitable for reliable focused ion beam (FIB)-assisted TEM lamellae preparation; therefore, only an ITO-wafer sample with its conductive silicon substrate is used for this analysis. Furthermore, XPS was performed for a pristine and a degraded ITO area on an ITO-wafer sample, which is presented and discussed in the Supporting Information in Figure S6 and Table S1. The results of the TEM investigation of a pristine and degraded ITO-wafer sample after 15 CV cycles are summarized in Figure 6. A dramatic change in the ITO layer structure caused by the electrochemical treatment can be observed. The thickness of the pristine ITO film measured directly via TEM is approximately 90 nm, which is in full consonance with values obtained by ellipsometry (see Table 1). This pristine, continuous layer has a polycrystalline structure with columnar grains; see Figure 6a. In contrast, the treated layer contains a number of round-shaped inclusions, which apparently are caused by the formation of metallic nanodroplets; see Figure 6b and the XPS data discussion in the Supporting Information. Most of the droplets occupy the top area of the layer. However, their coalescence and movement while CV cycling may lead to the local disruption of the whole film, inducing the appearance of voids and pores, which are clearly visible in Figure 6b (high-resolution images in the inset).

High-angle annular dark-field (HAADF) STEM imaging complemented with EDXS, as shown in Figure 6c,d, brings more insight into structural and chemical evolution. Note that EDXS is not sensitive to different oxidation levels of an element, and quantification of low atomic weight elements, such as oxygen, is difficult. Therefore, only atomic ratios of heavier elements, such as indium and tin, are considered to be significant. The atomic percentages are listed for oxygen, indium, and tin in Table 2 differentiating between the top region “T” and the bottom region “B” of the cross-section. From this, we learn that even the pristine ITO-wafer layer having a uniform structure as presented in Figure 6c is a graded layer. The In to Sn ratios are different at the top and bottom, being approximately 10 and 14, respectively. This indicates an increase of In or decrease of Sn content from top to bottom. Note that this material gradient is processing related and not a consequence of the electrochemical cycling, and it has no noticeable impact on the transparency. However, this finding can unambiguously explain the grading of the refractive index (and resistivity) revealed by ellipsometry, as presented in Subsection 2.1 and Figure 3a,b.

**Table 2:** Summary of EDXS (Cross-Section) Analysis of the Pristine and Treated (15 CV Cycles, Potential Range from −1.5 to +0.8 V) Areas of an ITO-Wafer Sample (*N* = 1)^a^

cross-section EDXS	O, at. %	In, at. %	Sn, at. %	In/Sn ratio
pristine “T”	42.0	53.0	5.0	10.5
pristine “B”	39.5	56.5	4.0	14.0
treated “T”	25.5	69.0	5.5	12.5
treated “B”	40.5	55.5	4.0	14.0

a “T” and “B” refer to the top and bottom regions of the ITO layer, respectively. Treated “T” covers the average result of the EDXS analysis of the droplet areas. The assessed EDXS measurement error is ±1.5 at. %.^88^ Note that sample-to-sample variations are also expected. Complementary XPS analysis of the specimen surfaces can be found in Table S1.

The brighter appearance of round-shaped particles in the HAADF image Figure 6d of the degraded ITO layer points to more higher atomic weight elements present in these regions and, hence, a partial loss of oxygen in this region. In the EDXS oxygen elemental map, the corresponding darker regions of nanodroplet form, i.e., O-depleted particles, are also clearly visible. Indeed, the atomic ratios change significantly in the top region of the treated sample compared with the pristine ITO layer; see Table 2 entries for pristine “T” and treated “T”. While the oxygen content largely drops, the In to Sn ratio rises slightly from about 10.5 to 12.5. These results are in good agreement with the XPS analysis; see Table S1. We do not consider this In/Sn ratio to play a determining part in the reductive darkening of the layer. The remaining detected oxygen content could also be overestimated. With respect to the size of the metallic nanodroplets (about 30 to 50 nm) and the thickness of the lamella of approximately 75 nm, this originates to some extent from a matrix with a higher residual O content surrounding the strongly reduced nanodroplet regions. Clearly, the top region of the ITO layer suffers from a reductive loss of oxygen, which causes darkening and a loss of visible transparency of the ITO layer.^22,27^ Reductive darkening due to the formation of oxygen vacancies, either processing- or operation-related, is also known for varying types of functional oxidic materials.^30−33,35,36^ Interestingly, the bottom region of the treated sample looks virtually intact, both structurally and chemically, even after 15 CV cycles. As for pristine ITO (entry pristine “B” in Table 2), the oxygen content is very similar and the In/Sn ratio remains unchanged at 14 (entry treated “B” in Table 2). This intact part of the film unless destroyed appears to be responsible for maintaining conductivity and, consequently, the repeatability of multiple CV cycles. On the contrary, the optical transparency is already progressively lost due to the reductive disruption and darkening of the ITO layer surface. These findings support and complement the conclusions on the ITO layer evolution during electrochemical treatment drawn from the operando ellipsometry investigation, as discussed in Subsection 2.2.

## 3 Conclusions

In this work, we present operando ellipsometry combining electrochemistry and spectroscopic ellipsometry as a versatile tool to monitor the evolution of the complex refractive index and the electrical properties under wet electrochemical conditions without interfering with electrochemical cycling. Conventional CCD-detection-based ellipsometers provide fast enough acquisition of Ψ and Δ spectra suitable for isotropic and uniaxial samples, also including graded layers, to acquire a significant number of full spectra during a CV cycle. This allows detailed monitoring of solid–liquid interfaces under operational conditions with a few seconds of time resolution.

The widely implemented transparent thin film electrode ITO (commercially available on glass substrate) serves as a model system to trace the reductive darkening in a pH-neutral, water-based electrolyte during electrochemical cycling. Since ITO is transparent in the visible and behaves metal-like in the infrared spectral range, it allows optical detection of the resistivity by a Drude-model of the IR-absorption of free charge carriers. ITO, like other functional oxidic materials, is prone to cathodic reduction. This is noticeable as a visible darkening when exceeding a critical negative voltage in reverse bias, which is characterized by the onset of hydrogen evolution in a water-based electrolyte. The most pronounced change in the Ψ spectrum happens at the negative potential turning point, which is associated with a shift of the UV-absorption onset into the visible spectral range (darkening), while the Drude-absorption tail (resistivity) basically remains unchanged. This means a certain loss of transparency while the conductivity is retained. In forward bias, the Ψ spectrum is partially but never fully restored, indicating the irreversibility of the degradation. While the darkening of the ITO layer is leaping forward with every CV cycle, the CV curves are reproducible (up to 30 cycles) due to retained conductivity.

Ellipsometric modeling is feasible only for the early stage of degradation (the first CV cycle) because the strongly increasing roughness of the ITO sample imposes complexity that cannot be handled by fitting of Ψ and Δ spectra. However, the qualitative evolution of the Ψ and Δ curves is still instructive to monitor over multiple CV cycles. They clearly indicate the potential range where degradation and partial recovery happen, and with this, they illustrate the incremental nature of the progress.

To visualize the nanomorphology and identify the chemical changes, we implement cross-sectional TEM-based techniques, including EDXS mapping supported by XPS analysis of the sample surfaces. We show the reductive (loss of oxygen) formation of metallic indium-enriched nanodroplets at the interface region in contact with the electrolyte. This disrupts the layer, allowing further penetration of the electrolyte for a proceeding reductive degradation down to the bottom of the ITO layer. Even after 15 CV cycles, there is still an intact ITO bottom layer that maintains the conductivity and allows continued CV cycling, while the reductive loss of oxygen already causes the transparency to decrease from more than 80% to less than 20% in the visible spectral range.

The operando ellipsometry approach is a general-purpose monitoring tool suitable for various fields of research, comprising solid–liquid interfaces and electrochemical activity, due to its sensitivity and nondestructive nature. We consider its application especially relevant for battery research since electrochemical cycling is the core purpose of battery electrodes. Operando ellipsometry can be used to trace failure points and is also valuable for controlling production processes involving intentional optical and electrical changes of functional thin film materials. Since ellipsometry is also sensitive to adsorbates, such as biomolecules, on plane surfaces and even at functionalized hybrid interfaces, it is also a versatile tool for (bio)-sensing purposes.

## 4 Materials and Methods

### 4.1 Sample Preparation

ITO on glass sample plates type XY15S were bought from Xinyan Technology LTD. (ITO-glass), cut into 25 × 25 mm^2^ pieces, and otherwise used as received. The ITO layer was coated onto 1.1 mm thick soda lime glass with a SiO_2_ surface layer; supplier specifications were (155 ± 20) nm layer thickness and less than 15 Ω sheet resistance.

ITO was sputter-coated onto (111)-Si-wafers with thermal oxide of (1009 ± 1) nm thickness (ITO-wafer). The thickness was determined by spectroscopic ellipsometry in reflection (J.A. Woollam M-2000 DI) using a model included within the CompleteEASE 6 software and further confirmed by cross-sectional TEM. The wafers were cut into approximately 25 × 25 mm^2^ pieces and piece-wise coated with ITO to avoid lateral thickness inhomogeneities. The coating of the silicon wafer pieces with ITO layers was performed as a sputter-down process in DC mode in the sputter module of a cluster coating system CS 730 ECS (von Ardenne Anlagentechnik). The plasma medium was pure Ar gas (Linde, purity of 6.0) mixed with pure O_2_ gas (Linde, purity 4.5). The total pressure in the sputter chamber was 3 × 10^–3^ mbar (PID-controlled). The O_2_ partial pressure was set by controlling the flow rates of Ar and O_2_ to 20 and 0.7 sccm, respectively. An ITO target with a composition of 90% In_2_O_3_ and 10% SnO_2_ bonded to a 200 mm round magnetron was used. The sputter plasma was generated in a DC process with 39 V and 2.5 A (generating a nominal power of 1 kW). The sputter race-track zone on the target has a diameter of approximately 100 mm, which causes a slight inhomogeneity of the layer thickness on the substrate. The substrate was heated from the backside with a radiative heater for 900 s before and for 600 s after the coating process.

### 4.2 Ambient and Operando Ellipsometry and Electrochemistry

A J.A. Woollam M-2000 DI single rotating compensator ellipsometer (PCSA) with a horizontal sample stage was used for ambient and operando ellipsometric measurements. For ambient measurements in reflection, angles of incidence (AOIs) from 45 to 75° in steps of 5° were chosen to record the spectroscopic ellipsometry (SE) data Ψ and Δ. The spectral range from 193 to 1690 nm was recorded with two CCD-detector arrays offering 705 wavelengths simultaneously with a bandwidth of 5 nm within the UV–vis and of 10 nm within the NIR spectral range. The vendor provided software, CompleteEASE, which was used for data recording (version 5) and analysis (version 6).

A multisample analysis (MSA) was used to combine multiple measurements from different samples to obtain a representative complex refractive index for a sample system. Only certain parameters were allowed to vary for each dataset. For ITO-glass, it was possible to include transmission intensity spectra for parameter decorrelation.^80^ Incoherent backside reflections (ITO-glass only, allowed to vary ranging from 0.9 to 1.6) as well as bandwidth limitations (5 nm for UV–vis switching to 10 at 1000 nm) have been accounted for in the SE data, and depolarization data have been included in the fitting. The fit weighting of the transmission data was increased to 1000% (ITO-glass only). For ITO-glass 3 pieces cut from different sample plates, each has been measured twice with a 45° azimuthal rotation, and these 6 SE scans are collectively fitted with 3 transmission intensity spectra (one per ITO-glass piece), i.e., *N* = 9. The refractive index of the 1.1 mm thick glass substrate has been determined in advance from a sample piece, where the ITO layer has been removed by etching with nascent hydrogen (Zn powder plus half-concentrated hydrochloric acid), using a Kramers–Kronig consistent B-Spline with transmission intensity data included. For the ITO-wafer, 6 pieces from different sputter-coating cycles have each been measured twice with a 45° azimuthal rotation, giving 12 SE scans for joint fitting (*N* = 12). The model for silicon wafers with thermal oxide included in CompleteEASE 6 was used to determine the SiO_2_ thickness to be (1009 ± 1) nm. After all fit parameters were turned off, this model was used as a substrate for the ITO-wafer samples.

All common (if not indicated by MSA) fit parameters for the batch analysis are summarized in Table 3 after multiple fitting iterations to minimize the MSE (mean square error). For ITO-wafer samples, multiple parameters were allowed to vary over the different SE measurements: thickness, roughness, resistivity, resistivity grading, and scattering time.

**Table 3:** Common Joint Active Fit Parameters for ITO-Glass (*N* = 9) and ITO-Wafer (*N* = 12) Multi-Sample Analysis Performed with CompleteEASE Version 6^a^

parameter	ITO-glass	ITO-wafer
MSE	11.6	35.7
thickness (nm)	110	88 ± 1 (MSA)
roughness (nm)	4.5	3.6 ± 0.4 (MSA)
UV pole amplitude (eV^2^)	345	56
UV pole energy (eV)	10.9	7.7
ϵ infinity offset	0.0	2.0
Tauc–Lorentz oscillator		
amplitude (eV^2^)	51.9	11.4
broadening (eV)	0.99	1.15
center energy (eV)	4.61	4.85
band gap energy (eV)	3.87	2.71
Gaussian oscillator		
amplitude (eV^2^)	2.0	2.4
broadening (eV)	2.58	1.76
center energy (eV)	6.21	6.22
Drude (RT) term		
resistivity (10^–4^ Ω cm)	1.7	2.9 ± 0.4 (MSA)
scattering time (fs)	7.5	4.7 ± 0.6 (MSA)
resistivity grading (%)		53 ± 15 (MSA)

a Rounded values are given for clearness since the parameters may still vary in their endmost decimal places without changing the MSE. MSA indicates that this parameter is varied for each dataset, mean and standard deviation are calculated manually outside CompleteEASE.

For both ITO-glass and ITO-wafer samples, the ITO layer was modeled with an isotropic oscillator model composed of a Tauc–Lorentz^89,90^ and a Gaussian oscillator to describe interband transitions within the UV spectral range plus a Drude term to account for the free carrier effects on the dielectric function, plus a surface roughness layer. According to the CompleteEASE manual, the surface roughness is modeled with a Bruggeman effective medium approximation (EMA) mixing the bulk layer (here, ITO) with 50% voids, i.e., lowering the refractive index of the bulk layer. Thickness is the only fit parameter that takes half of its thickness from the layer below (here, ITO bulk). Therefore, it is always a “stealing” layer. The surface roughness thickness can turn negative, which rather resembles an increase of the surface refractive index. The IR pole was set to zero and turned off so as not to interfere with the Drude term. This Drude (RT) term is derived from a Lorentz oscillator but with the center energy set equal to zero, where (RT) indicates in the CompleteEASE software that it has resistivity and scattering time as fit parameters. For free carrier optical absorption, there is a direct dependence of the absorption on the carrier density. With this, a possible vertical carrier concentration profile can be modeled with a graded multiple sublayer complex refractive index profile.^82^ Parametric linear grading allows the resistivity parameter of the Drude term to vary with depth through the ITO layer, which was sliced into 11 equally thick sublayers. The resistivity was found to be lower at the bottom of the film and increased by about 50% toward the top.

According to the CompleteEASE manual, the “ellipsometry”-resistivity ρ_(*E*)_ of a linear parametric graded layer is calculated from the multiple sublayers using the parallel-resistor model
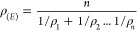
1where *n* is the number of sublayers implemented and ρ_*n*_ is the resistivity of the *n*th sublayer.

Alternatively, the sheet resistance *R*_S_ was measured using a four-wire sensing head (SP4) connected to a Keithley 2400 source measure unit to complement the data derived from ellipsometry. The “4-wire”-resistivity ρ_(4W)_ is calculated according to
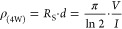
2where *d* is the layer thickness (here, determined from ellipsometry) and *V* and *I* are the measured voltage and current.

For fitting the operando ellipsometry scans, the ambient index was allowed in CompleteEASE to be larger than one. The refractive index of water included in the CompleteEASE database (originally taken from Palik’s database) was chosen to model the electrolytic environment. The backside of the ITO-glass samples was always wetted with the electrolyte before mounting the electrochemical cell to make sure that the electrolytic medium was the same on both the front and the backside of the sample. The above-discussed isotropic, nongraded oscillator model for ITO-glass was used as the starting model. However, the Gaussian oscillator was omitted for simplicity for modeling in an electrolytic environment. Screenshots of the CompleteEASE modeling showing all fit parameters as well as measured and fitted Ψ and Δ curves for the selected time slices shown in Figure 4 are collected in the Supporting Information in Figure S8.

The electrochemical cell was fabricated in our in-house workshop. It is mounted on top of a flat-lying sample and a rubber seal defines an oval-shaped area of (0.85 ± 0.05) cm^2^ that is exposed to the physiological electrolyte (see details below). The ellipsometric probe beam enters and exits through windows oriented normally to the beam. With this, the AOI on the sample surface is fixed at 70° for operando measurements. Due to the transparency of the water-based electrolyte, the spectroscopic window is limited to 225–1300 nm. The CCD-based detection provides full Ψ and Δ spectra every 2.63 s. All electrochemical measurements are synchronized with the ellipsometric measurements and are conducted in a three-electrode configuration using an IVIUM CompactStat potentiostat operated with the vendor-provided software IviumSoft. Copper tape with conductive glue (Conrad Electronics) is stuck to a corner of the ITO layer outside the electrolyte exposed area to act as a working electrode. A coiled loop of platinum wire with a 0.25 mm diameter is positioned a few millimeters above the sample through an opening on the side to serve as a counter electrode. Another feed-though dips a silver wire that has been electrochemically chlorinated in hydrochloric acid into the electrolyte to act as a Ag/AgCl pseudo-reference electrode. CV scans were started at 0 V in reverse bias direction after typically 8 s pretreatment for equilibration. The scan rate was 50 mV/s. The physiological electrolyte of choice is a HEPES-buffered Krebs–Ringer solution that consists of 140 mM sodium chloride, 5 mM potassium chloride, 1 mM magnesium chloride, 2 mM calcium chloride, 10 mM d-(+)-glucose, and 10 mM HEPES (4-(2-hydroxyethyl)-1-piperazineethanesulfonic acid) dissolved in deionized water. The pH was adjusted to 7.4 with a sodium hydroxide solution.

For all measurements with the electrochemical cell, the electrolyte contained a natural ambient amount of oxygen. Purging the electrolyte with an inert gas such as nitrogen could cause bubble formation, which would disrupt the ellipsometric measurements. Since all the electrochemical degradation reactions of the ITO layer are based on reduction, they are basically independent of the oxygen content, as shown in the Supporting Information in Figure S7.

### 4.3 Structural and Chemical Characterization

X-ray diffraction of pristine ITO samples was used to confirm the polycrystalline nature adopting a bixbyite cubic crystalline phase^91^ (see Supporting Information Figure S1).

The morphology of the ITO films was characterized in plan-view geometry and at 45° to the specimen’s surface using a Zeiss CrossBeam 1540 XB (Zeiss, Germany) SEM. SEM images of pristine material and specimens after electrochemical failure were collected at a 5 keV acceleration voltage by applying a secondary electron detector.

XPS measurements were additionally employed as quantitative chemical surface characterization and are presented and discussed in the corresponding section of the Supporting Information in Figures S5 and S6, as well as in Table S1.

TEM imaging was carried out in a JEOL JEM-2200FS (JEOL, Japan) operated at an acceleration voltage of 200 kV. The TEM is equipped with an in-column Omega filter and a TemCam-XF416 (TVIPS, Germany) CMOS-based camera. Snapshots were recorded applying zero-loss filtering. (HR)TEM data processing was performed with Gatan Microscopy Suite and JEMS simulation software. Due to methodological reasons, the ITO films, in this case, were sputter-coated onto silicon wafers with an insulating silicon dioxide surface layer. To trace material evolution, two site-specific cross-sectional lamellae were cut via FIB milling (CrossBeam 1540 XB) from the region affected by electrochemical treatment and from the neighboring area (pristine material). To avoid any detrimental sample illumination and keep the specimen surface intact, a 40 nm thick tungsten protection layer was formed with magnetron sputtering utilizing a Compact Coating unit CCU-010 HV (Safematic, Switzerland). Prior to lamellae cutting, the samples were covered first with an electron beam stimulated W deposit, followed by an ion-stimulated W sacrificial layer to additionally protect the surface. The FIB was operated at an acceleration voltage of 30 and 5 kV for sample lift-out and final thinning, respectively. EDXS analysis was performed in scanning (S)TEM mode for qualitative elemental characterization of the specimen’s cross-section with an X-Max^N^ 80 T detector from Oxford Instruments (UK). The data were processed with dedicated Aztec software (Version 4.1). The assessed measurement error was ±1.5 at. %.^88^

## Supporting Information Available

XRD pattern of pristine ITO; CV and Ψ scan of ITO-glass in potential window −2.0 to +1.0 V (4 cycles harsh treatment); CV and Ψ scan of ITO-wafer in potential window −2.0 to +0.8 V (4 cycles harsh treatment); plan-view and 45° view SEM images of pristine and harsh-treated ITO-glass samples; XPS survey spectra of ITO-glass; XPS survey and high-resolution spectra of ITO-wafer; and CV ITO-glass under inert N_2_ conditions (PDF)
Synchronized 1 cycle CV and Ψ scans ITO-glass sample, potential window −1.5 to +0.8 V (MP4)
Synchronized 15 cycles CV and Ψ scans ITO-glass sample, potential window −1.5 to +0.8 V (MP4)
Synchronized 15 cycles CV and Ψ scans ITO-wafer sample, potential window −1.5 to +0.8 V (MP4)
Synchronized 4 cycles CV and Ψ scans ITO-glass sample, potential window −2.0 to +1.0 V (harsh treatment) (MP4)
Synchronized 4 cycles CV and Ψ scans ITO-wafer sample, potential window −2.0 to +0.8 V (harsh treatment) (MP4)
