# Supplementary material for: Monitoring the Electrochemical Failure of Indium Tin Oxide Electrodes via Operando Ellipsometry Complemented by Electron Microscopy and Spectroscopy
Source: ACS Appl Mater Interfaces. 2024 Feb 7;16(7):9517–31. doi: 10.1021/acsami.3c17923 (PMC10895603; doi:10.1021/acsami.3c17923)
Supplement: Supplementary file 1 — am3c17923_si_001.pdf [file am3c17923_si_001.pdf]

SUPPORTING INFORMATION
Monitoring the Electrochemical Failure of Indium Tin Oxide Electrodes via Operando Ellipsometry complemented by Electron Microscopy and Spectroscopy

Alexey Minenkov,*,† Sophia Hollweger,‡ Jiri Duchoslav,† Otgonbayar Erdene-Ochir,‡,§ Matthias Weise,¶ Elena Ermilova,¶ Andreas Hertwig,¶ and Manuela Schiek*,‡

†*Christian Doppler Laboratory for Nanoscale Phase Transformations, Center for Surface- and Nanoanalytics (ZONA), Johannes Kepler University, A-4040 Linz, Austria*

‡*Center for Surface- and Nanoanalytics (ZONA), Institute for Physical Chemistry (IPC) & Linz Institute for Organic Solar Cells (LIOS), Johannes Kepler University, A-4040 Linz, Austria*

¶*Bundesanstalt für Materialforschung und -prüfung (BAM), FB 6.1 Oberflächenanalytik und Grenzflächenchemie, Unter den Eichen 44-46, D-12203 Berlin, Germany*

§*present address: Department of Chemistry, University of Cologne, Greinstr. 4-6, D-50939 Cologne, Germany*

E-mail: oleksii.minienkov@jku.at; manuela.schiek@jku.at

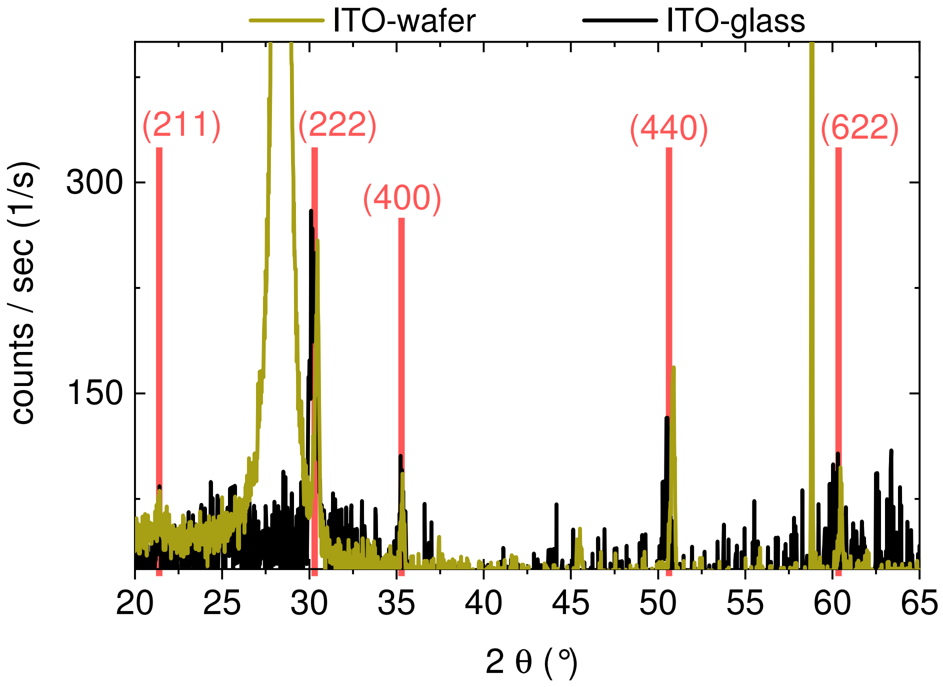

Figure S1: X-ray diffraction patterns of pristine ITO samples ($N = 1$) before electrochemical treatment: commercial ITO sample on glass XY15S bought from Xinyan Technology LTD (black line), sputter-coated sample on (1 1 1)-Si-wafer with thermal oxide of (1009 ± 1) nm thickness (green line). The massive signal around 28.5° is the (1 1 1)-plane of the Si-wafer substrate, which is accompanied by the higher order (2 2 2) peak, nominally at 57.0° but here shifted to 58.8°. For indexing of the peaks (Miller indexes in red) originating from the ITO layer, a calculated reference powder pattern from the $In_{1.875}O_3Sn_{0.125}$ crystallographic data ICSD 50848 was used.[1,2] All ITO layers are very similar and they are polycrystalline with random orientation of grains adopting the bixbyite cubic crystalline phase.

We thank Dr. Munise Cobet (IPC & LIOS, JKU) for performing XRD experiments (PANalytical X'Pert PRO, PIXcel 1D detector, monochromated Cu-$K\alpha$-radiation).

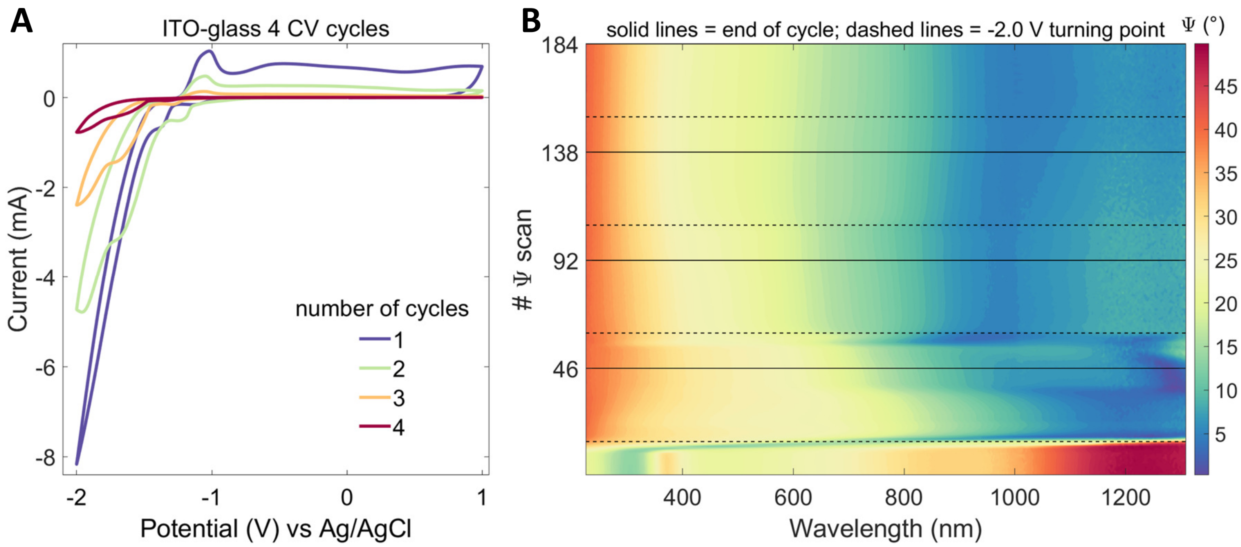

Figure S2: (a) Four CV cycles of an ITO-glass sample ($N = 1$) in Krebs-Ringer electrolyte starting from 0 V down to -2.0 V and up to $+1.0$ V measured against an Ag/AgCl pseudo-reference electrode with a scan rate of 50 mV/s. The CV changes significantly during this harsh electrochemical cycling, and after 4 cycles no current is flowing indicating a full destruction of the ITO-layer. The corresponding Ψ spectra (AOI = 70°) shown in (b) change markedly at negative bias around the turning point at -2.0 V already during the first CV cycle. The potential turning point from reverse to forward sweep is indicated by the dashed lines, and the solid lines indicate the end of a CV cycle at 0 V. Per CV cycle 46 Ψ spectra are recorded, giving 184 Ψ spectra in total. They are shown in a two-dimensional representation where the Ψ value in ° is color-coded. A video clip showing synchronized animated CV cycles and Ψ scans is included in the Supporting Information as Movie S4.

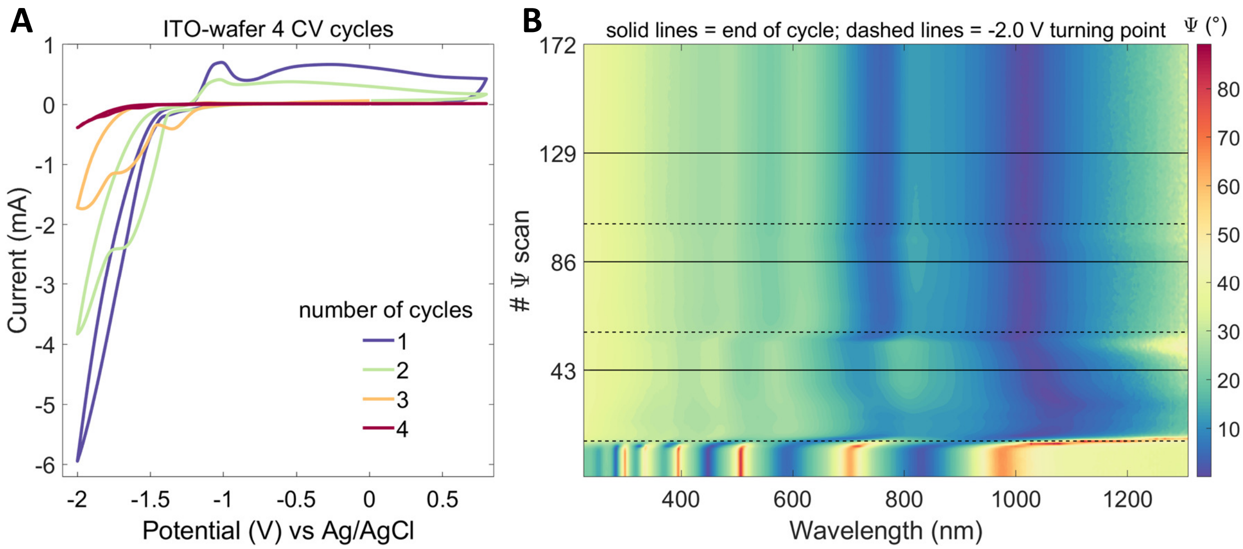

Figure S3: (a) Four CV cycles of an ITO-wafer sample ($N = 1$) in Krebs-Ringer electrolyte starting from 0 V down to -2.0 V and up to $+0.8$ V measured against an Ag/AgCl pseudo-reference electrode with a scan rate of 50 mV/s. The CV changes significantly during this harsh electrochemical cycling, and after 4 cycles no current is flowing indicating a full destruction of the ITO-layer. The corresponding Ψ spectra (AOI $= 70°$) shown in (b) change markedly at negative bias around the turning point at -2.0 V already during the first CV cycle. The potential turning point from reverse to forward sweep is indicated by the dashed lines, and the solid lines indicate the end of a CV cycle at 0 V. Per CV cycle 43 Ψ spectra are recorded, giving 172 Ψ spectra in total. They are shown in a two-dimensional representation where the Ψ value in ° is color-coded. A video clip showing synchronized animated CV cycles and Ψ scans is included in the Supporting Information for download as Movie S5.

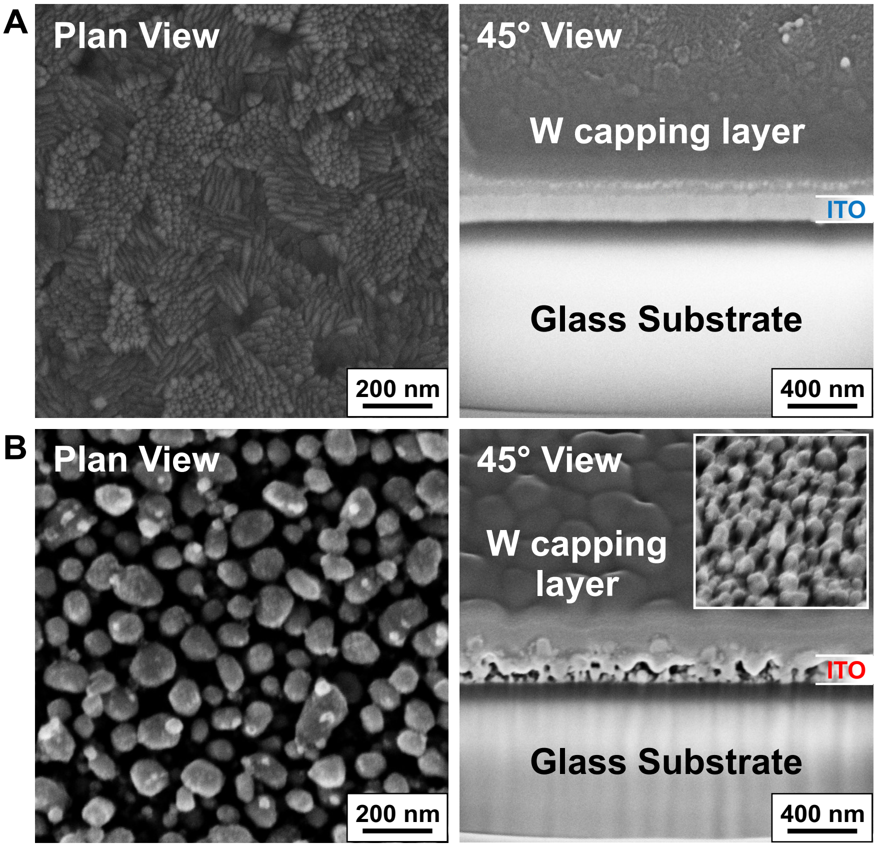

Figure S4: SEM images showing plan and cross-sectional (45° view) morphology evolution of the specimen on glass: (a) pristine continuous ITO layer, (b) discontinuous greatly reduced ITO after only 4 cycles of harsh treatment from −2.0 V to +1.0 V. The sample represents individual particles with a median size of approximately 100 nm. Distinct gaps between particles lead to a complete loss of conductivity. Cross-sectional cuts were performed via FIB using W as a protection layer.

X-Ray Photoelectron Spectroscopy (XPS) Characterization

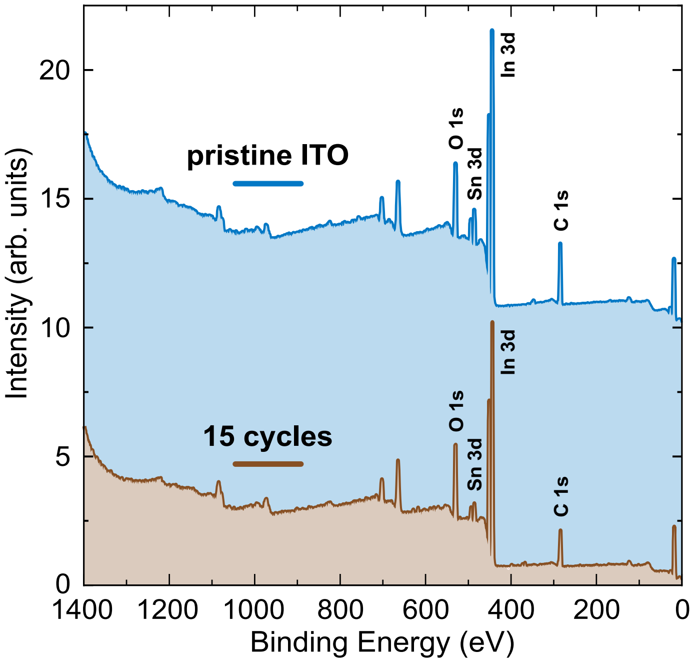

Figure S5: XPS survey spectra of a pristine ITO-glass sample and the same sample after 15 cycles of treatment from −1.5 V up to +0.8 V ($N = 1$). Note an increased In/Sn ratio in the treated specimen.

To assess the chemical elements present and their ratios, the surfaces of a pristine ITO-glass sample and a treated specimen were analyzed with XPS. The corresponding survey spectra are shown in Figure S5. As the surface of all specimens was contaminated with residuals including carbon, the In/Sn ratio is a more dependable reference to trace chemical changes in the specimens during treatment. The evaluation consistently shows a rise in In to Sn ratio for increasing electrochemical treatment time. Thus, in pristine ITO this ratio equaled 8.5 changing to 10.5 and further to 18 after 1 and 15 cycles, respectively. Considering the exclusive surface sensitivity of XPS, this result indicates that the nanoparticles formed due to ITO reduction consist almost exclusively of In. At the same time, a lack of spatial resolution of XPS allows for obtaining only composition data integrated over an area of hundreds μm^2. This limitation rules out dependable chemical analysis of inhomogeneities at the nanoscale and necessitates the application of more site-specific characterization methods

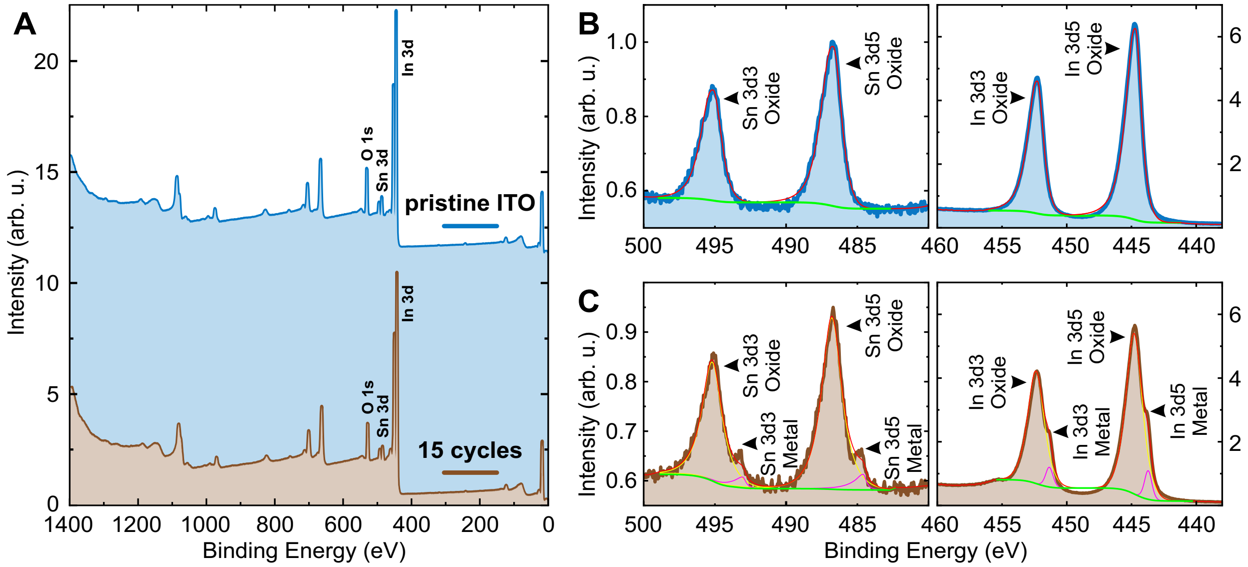

Figure S6: (a) Survey XPS spectra of a pristine ITO area on an ITO-wafer sample, and a degraded area on the same sample after 15 cycles of electrochemical treatment in the potential window from −1.5 V to +0.8 V in a physiological electrolyte. High-resolution spectra of (b) the pristine ITO-wafer sample area and (c) the treated specimen. The fitting of metallic peaks in (c) is depicted with a magenta line. The green lines in the high-resolution XPS spectra represent the background subtraction used to evaluate the peaks.

such as cross-sectional TEM-based techniques, see main manuscript Figure 6 and Table 2.

To quantitatively evaluate the chemical evolution of the ITO layer caused by electrochemical treatment, XPS was also performed for a pristine and a degraded ITO area on an ITO-wafer sample after conducting 15 CV cycles in the potential range from −1.5 V to +0.8 V in a physiological electrolyte. To improve evaluation quality an approximately 5 nm thick surface layer had been removed *in situ* via Ar sputtering to minimize the impact of the residual contamination such as carbon. Survey spectra together with high-resolution spectra of In and Sn are presented in Figure S6(a)-(c). A summary of the XPS data can be found in Table S1. The high-resolution spectra in Figure S6(b) show that in the pristine ITO layer both Sn (left side plot) and In (right side plot) are in their fully oxidized, non-metallic states (Sn(IV) and In(III)). Likewise, the atomic percentages found for oxygen, indium and tin being roughly 60 at%, 40 at%, and 4 at%, respectively, indicate fully stoichiometric oxides that are In_2O_3 and SnO_2. We could not find reliable evidence for oxygen vacancies and

sub-stoichiometric oxides within our analysis, even though these are widely discussed in literature.[3–5] In contrast, the spectra of the electrochemically treated ITO layer in Figure S6(c) exhibit additional peaks at 493 eV and 485 eV (left side plot), and at 466.5 eV and 452.5 eV (right side plot) visible as shoulders corresponding to metallic Sn and In, respectively.[3] From the ratios of metallic and oxidic species, see Table S1, we evaluate that about 8 % to 10 % of In and Sn are present in metallic form in the electrochemically degraded sample. The oxygen content is lowered accordingly. Furthermore, the In to Sn ratio (counting both metallic and oxidic species) increases from almost 10 in the pristine sample to 13 after electrochemical treatment confirming the accumulation of indium in the surface region.

Table S1: Summary of XPS (plan-view surface "S", Ar-sputtered approx. 5 nm deep) analysis of the pristine and treated (15 CV cycles, potential range from −1.5 V to +0.8 V) area of the ITO-wafer sample ($N = 1$). The atomic percentages are rounded to one decimal place, because we expect a sample-to-sample variation of at least 0.5 % of the element's atomic ratios, which exceeds the estimated experimental error of the XPS measurement.

plan-view XPS	O, at% (oxide)	In, at% (oxide)	In, at% (metal)	Sn, at% (oxide)	Sn, at% (metal)	In/Sn ratio
Pristine "S"	59.9	36.4	-	3.8	-	9.7
Treated "S"	56.5	37.4	3.0	2.9	0.3	13.0

XPS analysis was performed with a Nexsa G2 system (Thermo Scientific, CZ). The samples were probed with monochromated Al-$K\alpha$-radiation (1486.6 eV) focused into a spot of 400 µm in diameter to ensure a good signal-to-noise ratio. Survey spectra were acquired with a pass energy of 200 eV and a binding energy (BE) step of 1 eV, whereas for the high-resolution spectra, a pass energy of 20 eV with a BE of 0.05 eV was utilized. A dual flood gun was used to compensate for charges accumulated on the surface, employing low-energy electron and Ar ion beams. The measured spectra were corrected with respect to the C1s peak of the adventitious carbon at 285.0 eV. The spectra were evaluated using the Avantage software package (Version 5.99) from the device manufacturer.

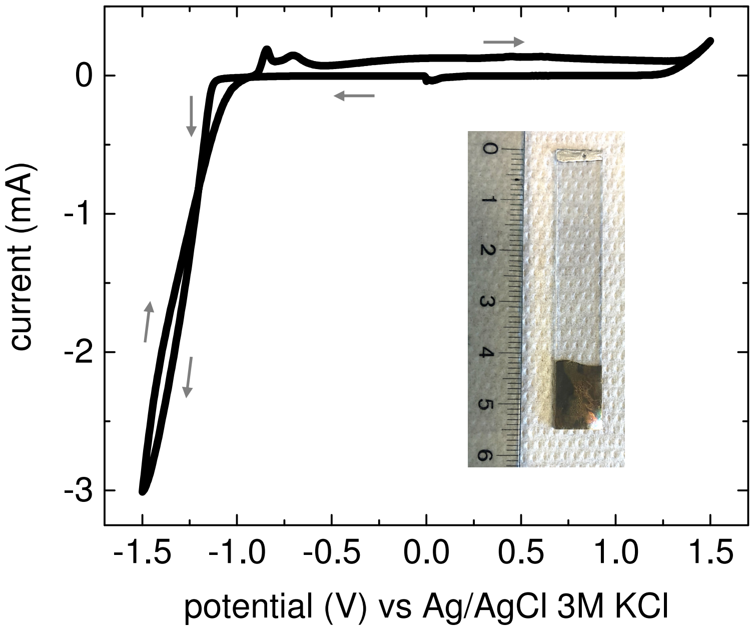

Figure S7: Cyclic voltammetry with a scan rate of 20 mV/s measured on an ITO-glass sample in pH-neutral HEPES-buffered Krebs-Ringer electrolyte after bubbling with nitrogen for 1 hour. A three-electrode setup in a 20 mL vial with Pt counter electrode, Ag/AgCl 3M KCl reference electrode and ITO working electrode connected to an Ivium CompactStat was used. The gray arrows mark the scan direction starting at zero potential. Note the crossing of the I-V curve at around −1.2 V. The ITO layer becomes chemically unstable at more negative potentials, in addition, hydrogen evolution occurs. Despite no indium or tin reduction peak is noticeable under reverse bias at a negative potential, under forward bias but yet at negative potential two peaks appear indicating indium / tin re-oxidation.[6] Note the absence of a peak indicating oxygen to peroxide reduction[7] under these inert nitrogen conditions, which appear in cyclic voltammetry scans in reverse bias at around −1.3 V under ambient conditions as shown in the main manuscript. However, the darkening due to the formation of metallic indium and tin happens likewise in nitrogen conditions as pictured in the inset.

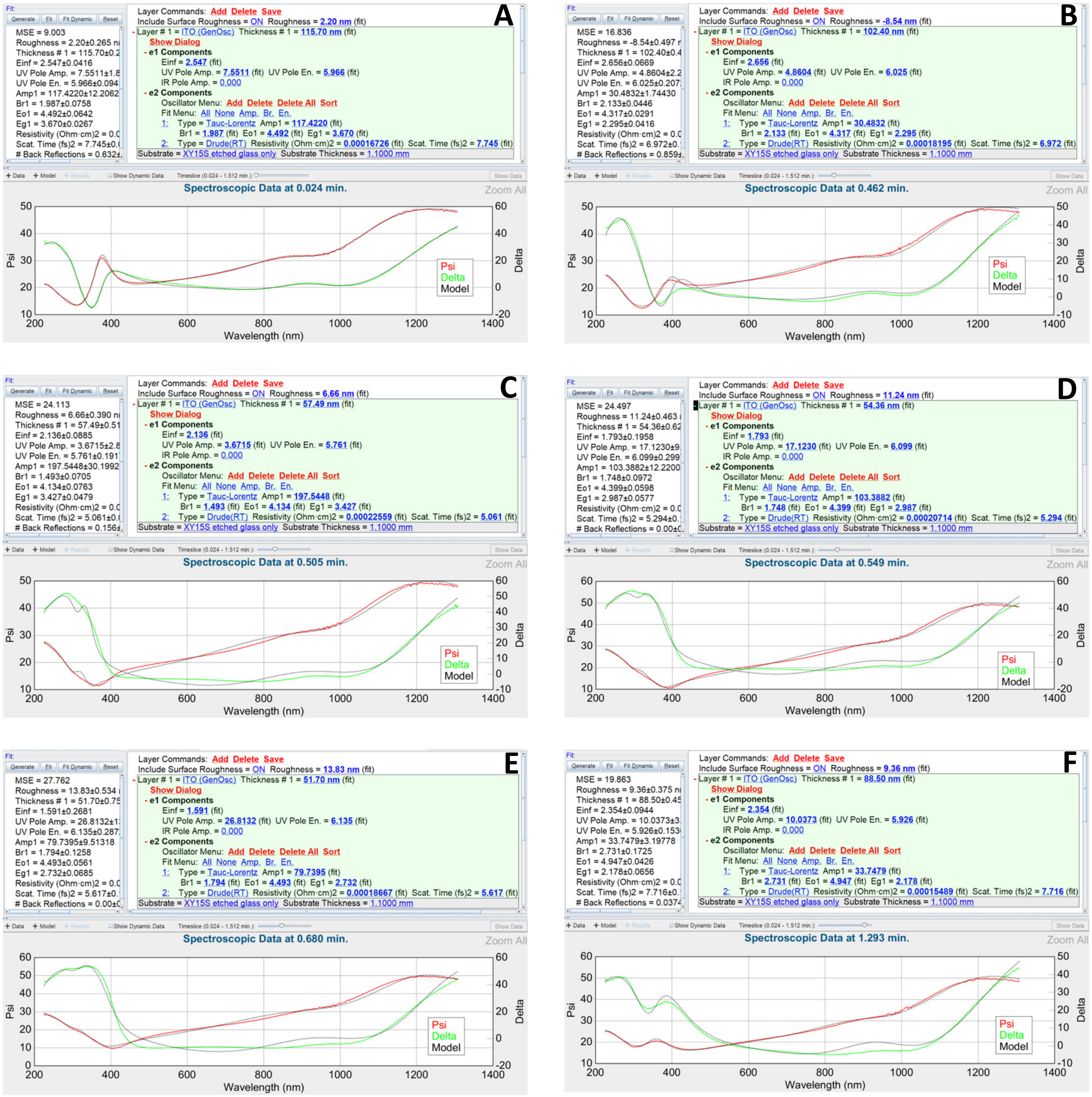

Figure S8: Screenshots from CompleteEASE version 6 showing the fits of the *operando* ellipsometry scans of an ITO-glass sample ($N = 1$) for a single CV cycle for the selected time slices as discussed in the main manuscript in Figure 4 (color refers to the main manuscript): (a) 1.4 s (dark blue lines), (b) 27.7 s (light blue lines), (c) 30.3 s (green lines), (d) 32.9 s (ocher lines), (e) 40.8 s (light brown lines), (f) 77.6 s (dark brown lines). Due to the transparency of the water-based electrolyte, the spectroscopic window is limited to 225 nm to 1300 nm. Ambient Index > 1 using water refractive index from the CompleteEASE data base (originally from Palik's database) on both the front and the backside of the sample. Wetting of the ITO-glass samples with electrolyte before mounting to the electrochemical cell was ensured. The oscillator model obtained for ITO-glass in air conditions was used as starting model, but omitting the Gaussian oscillator for simplicity.

References

(1) https://www.ccdc.cam.ac.uk/structures/Search?Ccdcid=50848&DatabaseToSearch=ICSD, accessed 29.01.2023.

(2) Nadaud, N.; Lequeux, N.; Nanot, M.; Jové, J.; Roisnel, T. Structural Studies of Tin-doped Indium Oxide (ITO) and $In_4Sn_3O_{12}$. *J. Solid State Chem.* **1998**, *135*, 140–148.

(3) Liu, L.; Yellinek, S.; Valdinger, I.; Donval, A.; Mandler, D. Important Implications of the Electrochemical Reduction of ITO. *Electrochim. Acta* **2015**, *176*, 1374–1381.

(4) Peng, S.; Cao, X.; Pan, J.; Wang, X.; Tan, X.; Delahoy, A. E.; Chin, K. K. X-ray Photoelectron Spectroscopy Study of Indium Tin Oxide Films Deposited at Various Oxygen Partial Pressures. *J. Electron. Mater.* **2016**, *46*, 1405–1412.

(5) Tsai, T.-M.; Tan, Y.-F.; Wu, C.-H.; Yang, C.-C.; Chen, W.-C.; Lin, C.-C.; Wu, P.-Y.; Zhang, Y.-C.; Chou, S.-Y.; Chen, Z.-Y.; Lin, T.-H. Impact of Oxygen Flow Rate on Performance of Indium-Tin-Oxide-based RRAMs. *J. Phys. D: Appl. Phys.* **2021**, *54*, 295103.

(6) Benck, J. D.; Pinaud, B. A.; Gorlin, Y.; Jaramillo, T. F. Substrate Selection for Fundamental Studies of Electrocatalysts and Photoelectrodes: Inert Potential Windows in Acidic, Neutral, and Basic Electrolyte. *PLoS ONE* **2014**, *9*, e107942.

(7) Jung, E.; Shin, H.; Antink, W. H.; Sung, Y.-E.; Hyeon, T. Recent Advances in Electrochemical Oxygen Reduction to H_2O_2: Catalyst and Cell Design. *ACS Energy Lett.* **2020**, *5*, 1881–1892.
